# Gene enrichment and co-expression analysis shed light on transcriptional responses to *Ralstonia solanacearum* in tomato

**DOI:** 10.1186/s12864-023-09237-0

**Published:** 2023-03-29

**Authors:** Jianlei Shi, Deju Shui, Shiwen Su, Zili Xiong, Wenshan Zai

**Affiliations:** 1 Southern Zhejiang Key Laboratory of Crop Breeding, Wenzhou Vocational College of Science and Technology, Wenzhou, Zhejiang 325006 China; 2 Fujian Provincial Key Laboratory of Crop Breeding by Design, College of Agriculture, Fujian Agriculture and Forestry University, Fuzhou, Fujian 350002 China

**Keywords:** Tomato, *Ralstonia solanacearum*, Differentially expressed genes, Gene annotation, Gene expression

## Abstract

**Background:**

Tomato (*Solanum lycopersicum*) is both an important agricultural product and an excellent model system for studying plant-pathogen interactions. It is susceptible to bacterial wilt caused by *Ralstonia solanacearum* (Rs), and infection can result in severe yield and quality losses. To investigate which genes are involved in the resistance response to this pathogen, we sequenced the transcriptomes of both resistant and susceptible tomato inbred lines before and after Rs inoculation.

**Results:**

In total, 75.02 Gb of high-quality reads were generated from 12 RNA-seq libraries. A total of 1,312 differentially expressed genes (DEGs) were identified, including 693 up-regulated and 621 down-regulated genes. Additionally, 836 unique DEGs were obtained when comparing two tomato lines, including 27 co-expression hub genes. A total of 1,290 DEGs were functionally annotated using eight databases, most of which were found to be involved in biological pathways such as DNA and chromatin activity, plant-pathogen interaction, plant hormone signal transduction, secondary metabolite biosynthesis, and defense response. Among the core-enriched genes in 12 key pathways related to resistance, 36 genotype-specific DEGs were identified. RT-qPCR integrated analysis revealed that multiple DEGs may play a significant role in tomato response to Rs. In particular, Solyc01g073985.1 (NLR disease resistance protein) and Solyc04g058170.1 (calcium-binding protein) in plant-pathogen interaction are likely to be involved in the resistance.

**Conclusion:**

We analyzed the transcriptomes of both resistant and susceptible tomato lines during control and inoculated conditions and identified several key genotype-specific hub genes involved in a variety of different biological processes. These findings lay a foundation for better understanding the molecular basis by which resistant tomato lines respond to Rs.

**Supplementary Information:**

The online version contains supplementary material available at 10.1186/s12864-023-09237-0.

## Background

Bacterial wilt is a devastating soil-borne vascular disease caused by *Ralstonia solanacearum* (Rs). As one of the most important pathogenic bacteria in plants, Rs can invade from plant wounds or secondary root caps, resulting in damage to vascular bundles that eventually reduces water transport and causes wilting and death. Tomato (*Solanum lycopersicum*) is grown worldwide and represents an important vegetable crop in many countries. However, due to continuous cropping and high temperature and high humidity, bacterial wilt in tomato-producing areas in China has become increasingly destructive, now representing the primary problem in tomato production. Plants face a variety of different biotic stresses, and have therefore developed sophisticated mechanisms to overcome them. Resistance genes (R genes), such as receptor-like proteins (RLPs), receptor-like kinases (RLKs), and nucleotide binding site-leucine rich repeats (NBS-LRRs), play a major role in recognizing and resisting pathogen invasion.

With the rapid development and widespread application of next-generation sequencing (NGS), transcriptome sequencing has proven to be an effective strategy to study the regulation of gene expression. RNA sequencing (RNA-seq) has been used to study a variety of different processes in tomato, including responses to biotic and abiotic stress, leading to the identification of genes involved in defense, signal transduction, and secondary metabolism. In an earlier analysis of the response of tomato to bacterial wilt, Ishihara et al. [1] found that 140 genes were up-regulated in the resistant tomato line LS-89 during Rs infection, including several β-1, 3-glucanase encoding genes. French et al. [2] found that, when compared to a susceptible line, defense genes in the root of the resistant tomato line Hawaii7996 were induced earlier and more strongly, and the auxin signaling pathway was suppressed. Zuluaga et al. [3] found a large number of new transcripts were induced in resistant potato during Rs infection, and the hormone-related genes were prominent in the susceptible line. Baichoo et al. [4] identified 19 differentially expressed genes (DEGs) in response to Rs invasion in different tomato lines and verified 6 of them in the other 5 *Solanum* species, with a close association found between gene expression changes and disease phenotype. Chen et al. [5] found that 1,137 genes were up-regulated and 9,048 genes were down-regulated in resistant eggplant inoculated with Rs, while the corresponding number of DEGs in susceptible eggplant was 6,087 and 5,832, respectively. Wang et al. [6] conducted transcriptome sequencing of resistant mutants and susceptible wild-type inoculated with Rs and identified multiple up-regulated genes in tobacco. Real-time quantitative PCR (RT-qPCR) analysis showed that the expression of *Ntab0876820* and *Ntab0833810* in two resistant mutants increased as infection progressed, while wild-type plants showed no such change.

In this study, RNA-seq technology was used to identify genes which were differentially expressed in response to Rs infection in both resistant and susceptible tomato lines. Functional annotation of the DEGs elucidated several key changes that lay a foundation for better understanding the Rs resistance response in tomato.

## Results

### Overview of mRNA libraries

A total of 75.02 Gb of clean reads were generated from the 12 libraries after removing reads with adapter contamination and low-quality bases. Clean reads of each library ranged from 5.73 Gb to 7.18 Gb with an average of 6.25 Gb. GC content was between 43% and 44% and the percentage of bases above Q30 was between 94% and 95%. The clean reads had alignment rates against the reference genome between 95% and 96% **(**Table 1**)**.

**Table 1:** RNA sequencing data statistics of tomato libraries

Libraries	Clean reads	Clean bases (Gb)	GC content (%)	Q30 (%)	Mapped reads
RC2	40,564,140	6.07	43.72	94.82	38,673,468
RC13	46,173,674	6.90	43.73	94.81	43,965,762
RC14	48,085,886	7.18	43.79	95.01	45,679,708
RT6	45,753,134	6.83	43.74	94.56	43,520,979
RT17	41,260,090	6.17	43.27	94.97	39,471,364
RT18	39,092,480	5.84	43.79	94.61	37,279,419
SC4	41,490,160	6.21	44.17	94.98	39,648,351
SC15	43,627,624	6.51	44.10	94.40	41,661,217
SC16	38,440,746	5.74	43.78	94.85	37,020,893
ST8	38,380,600	5.73	43.97	94.13	36,769,537
ST19	39,741,206	5.94	43.78	94.78	38,083,435
ST20	39,487,202	5.90	44.09	94.57	37,682,695

R and S represent resistant and susceptible tomato lines, respectively. C and T represent before and after *R. solanacearum* inoculation (*Rs*I), respectively. The numbers following these letters represent sample replicates. The same is below.

### Determination of differentially expressed genes

**Fig. 1:**
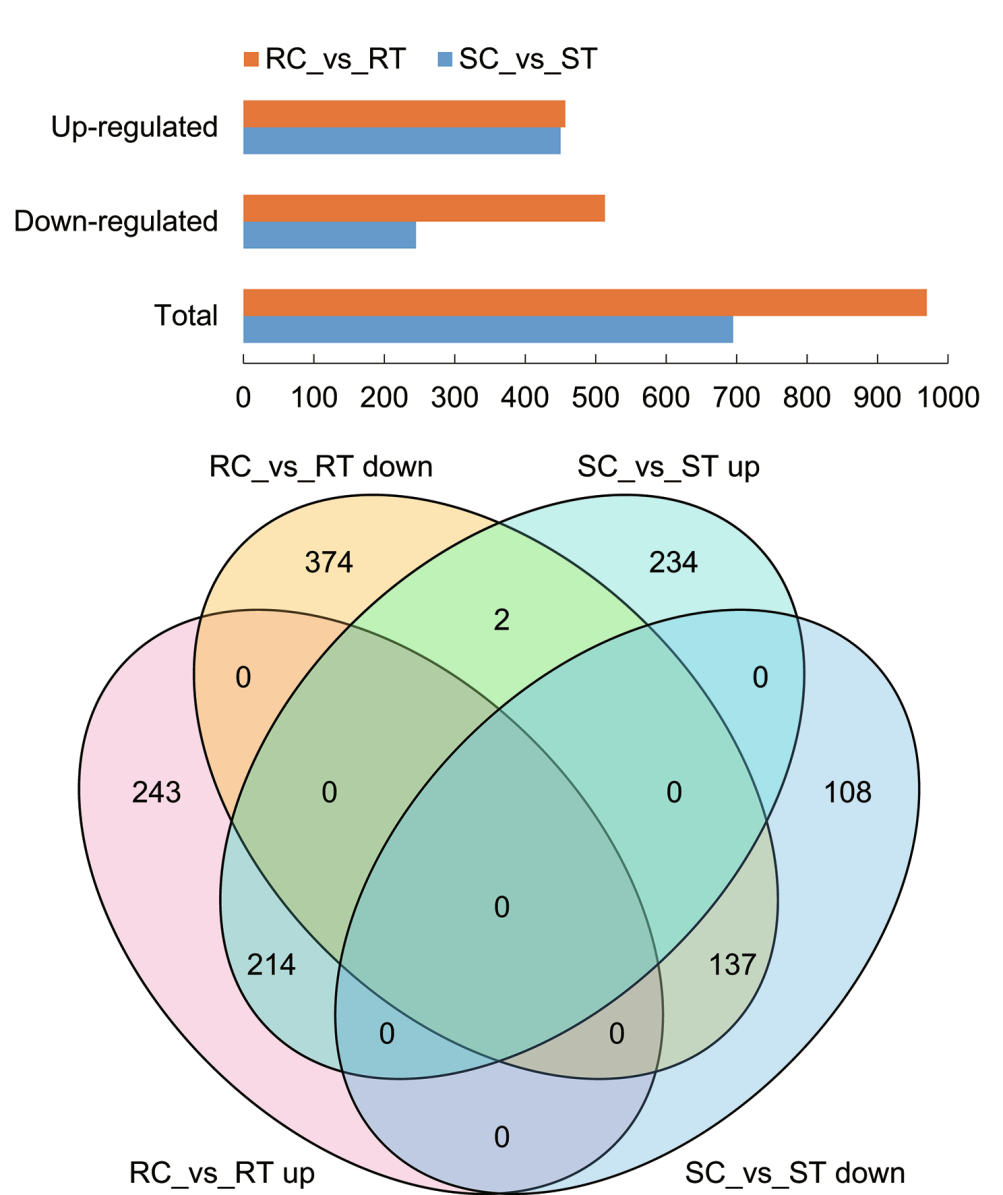
Barplot and venn diagram of differentially expressed genes. Up and down represent up-regulated and down-regulated gene expression, respectively. The same is below

A total of 35,371 genes were analyzed in this study, of which 31,903 (90.20%) were functionally annotated. In addition to the existing transcriptome, 1,296 new genes were assembled based on the RNA-seq analysis, accounting for 3.66% of the total. After log_2_ fold change (FC) and false discovery rate (FDR) filtering, 970 and 695 DEGs were identified in resistant and susceptible tomato lines, respectively, totaling 1,312 (including 32 new genes), accounting for 3.71% of the total genes in tomato. The number of up-regulated genes was 457 and 450 in resistant and susceptible tomato lines, respectively, and the number of down-regulated genes was 513 and 245, respectively **(**Fig. 1**)**.

The expression of *Solyc04g074440.1* and *Solyc12g096780.2* was down-regulated in the resistant line and up-regulated in the susceptible line upon inoculation. The number of genes up-regulated in the resistant line and unchanged in the susceptible line was 198, and vice versa 216. The number of genes down-regulated in the resistant line and unchanged in the susceptible line was 316, conversely 104. The total number of genotype-specific DEGs, which were uniquely differentially expressed in resistant or susceptible lines, was 836 (including 18 new genes) **(**Fig. 1and Table S1). *Solyc01g080460.3*, *Solyc07g008210.4*, and *Solyc08g075570.4* had a high level of expression with fold changes ranging from 8 to 32 in the resistant line, while that of *Solyc06g071830.2* and *Solyc08g075570.4* was 8–16 in the susceptible line.

### Differentially expressed genes were found to be involved in key pathways

**Fig. 2:**
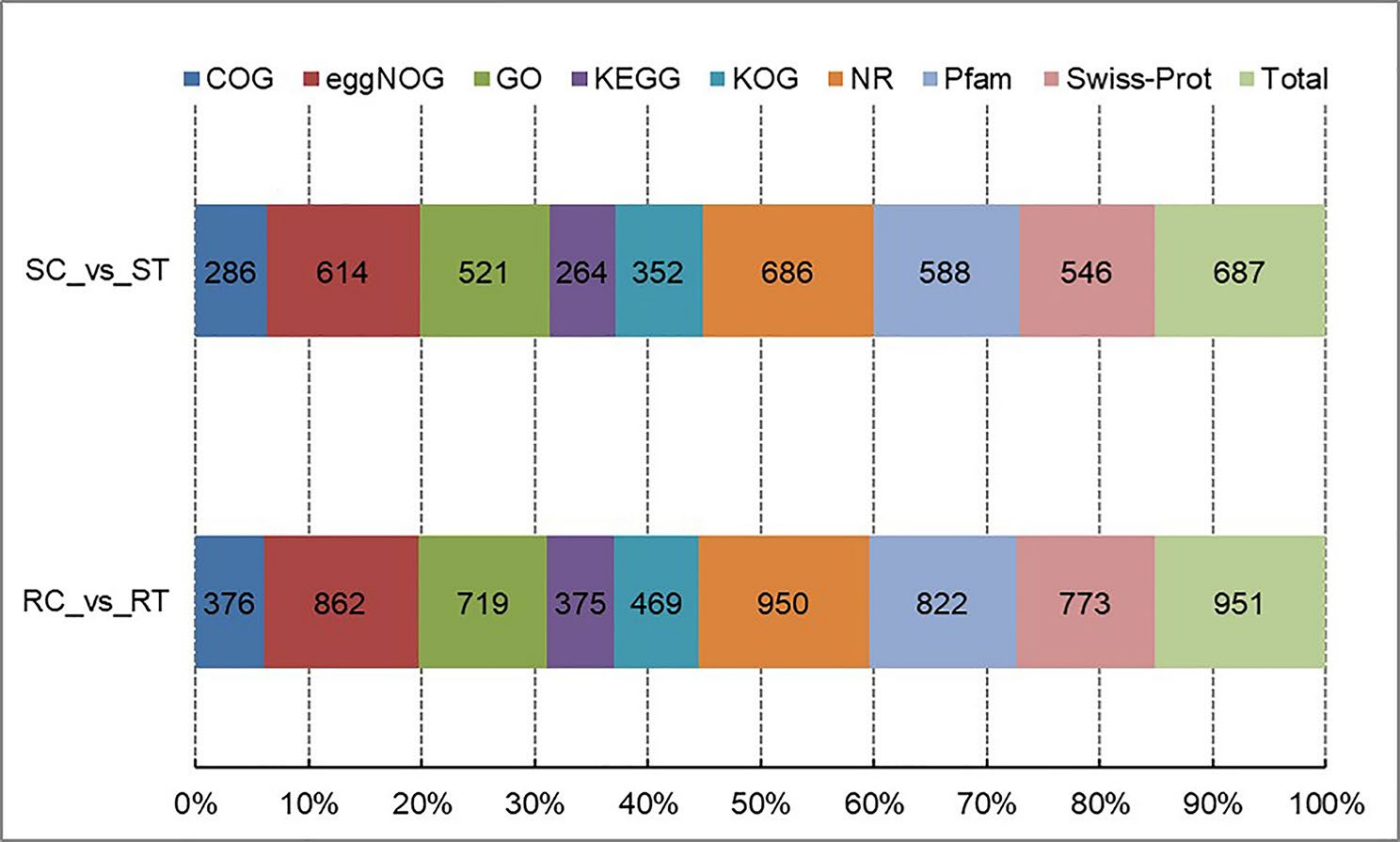
Annotations of differentially expressed genes in the eight databases

There were 951 and 687 DEGs in comparisons involving resistant and susceptible lines, respectively, 98.32% (1,290) of which were functionally annotated by comparison to eight major databases **(**Fig. 2**)**.

**Fig. 3:**
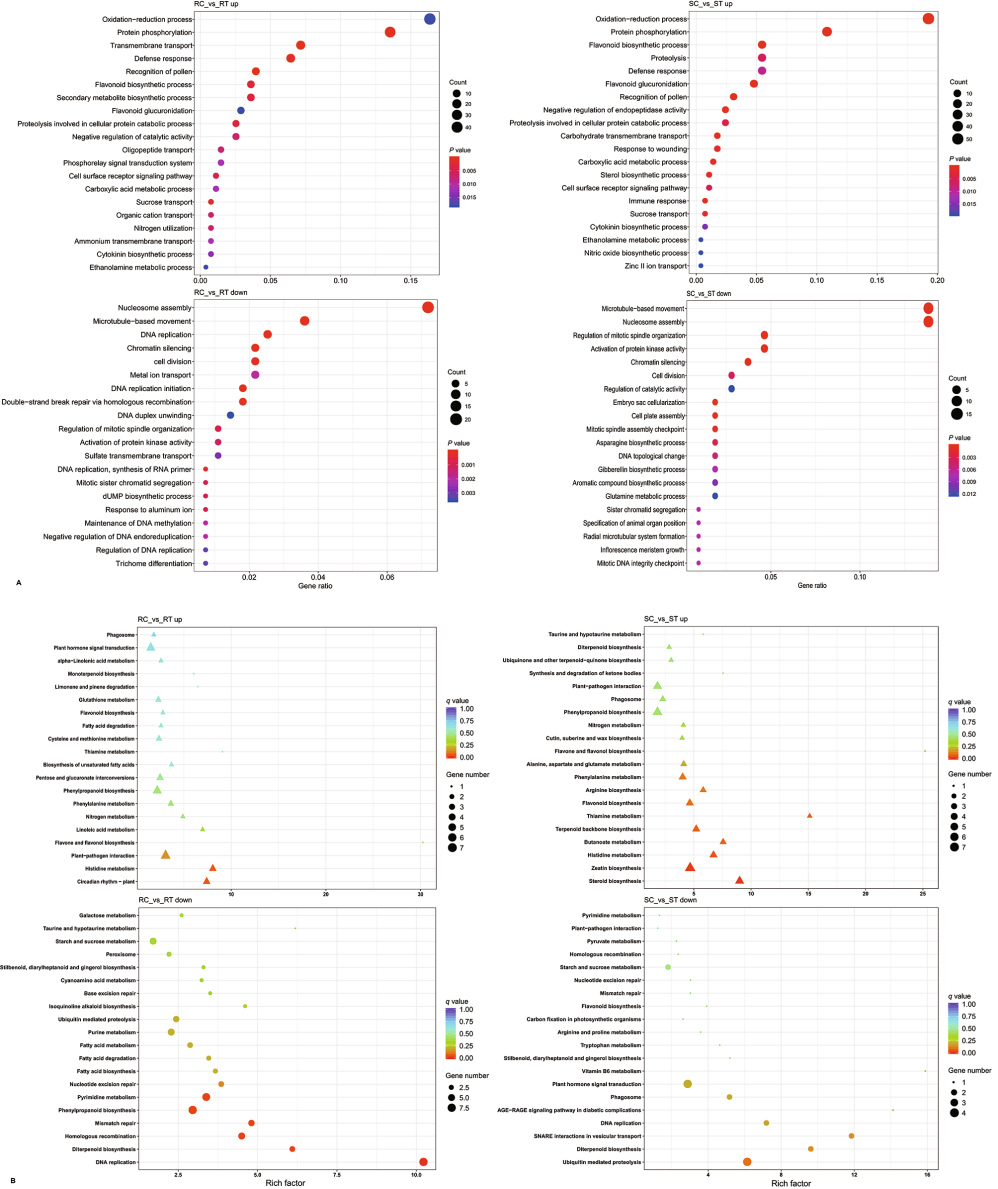
GO (**A**) and KEGG (**B**) enrichment of differentially expressed genes. The ordinate indicates biological processes (A) or metabolic pathways (B), and the name of which was shown on the left legend. Each circle or triangle represents a biological process (A) or metabolic pathway (B), and different colors and sizes represent (*P*) *q*-values and gene numbers, respectively. The rich factor indicates the significant enrichment of DEGs in a pathway. The *P* (*q*)-value indicates the reliability of enrichment significance

A total of 973 (74.16%) DEGs were annotated with gene ontology (GO) terms, which belonged to 47 functional groups. These groups were further divided into three categories: biological process (BP, 19 subclasses), cellular component (CC, 15 subclasses), and molecular function (MF, 13 subclasses). Up-regulated genes from the resistant tomato line were primarily involved in biological processes such as protein phosphorylation (GO:0006468, 38 DEGs), transmembrane transport (GO:0055085, 20 DEGs), defense response (GO:0006952, 18 DEGs), secondary metabolite biosynthesis (GO:0044550, 10 DEGs), and oxidation-reduction (GO:0055114, 46 DEGs). Down-regulated genes were mostly involved in DNA and chromatin activities. Up-regulated genes of the susceptible tomato line were primarily involved in flavonoid metabolism (GO:0009813, 16 DEGs) and oxidation-reduction process (GO:0055114, 57 DEGs), while the down-regulated genes were mostly involved in aromatic compound biosynthesis (GO:0019438) as well as chromatin activities **(**Fig. 3A**)**.

A total of 209 (15.93%) DEGs were annotated to 88 Kyoto encyclopedia of genes and genomes (KEGG) metabolic pathways, which were divided into the cellular process, environmental information processing, genetic information processing, metabolism, and organismal system. According to the *q*-value and rich factor, the top 20 most significantly enriched metabolic pathways were obtained **(**Fig. 3B**)**. Up-regulated genes of the resistant tomato line were primarily involved in the regulation of histidine metabolism (4 DEGs), circadian rhythm (4 DEGs), plant-pathogen interaction (7 DEGs), flavonoid biosynthesis (1 DEG), and linoleic acid metabolism (2 DEGs). Phenylalanine metabolism (3 DEGs), phenylpropanoid biosynthesis (6 DEGs), cysteine and methionine metabolism (3 DEGs), glutathione metabolism (3 DEGs), α-linolenic acid metabolism (2 DEGs), and plant hormone signal transduction (6 DEGs) were also significantly enriched. Down-regulated genes were mostly associated to DNA activity and phenylpropanoid biosynthesis (9 DEGs), in addition to fatty acid metabolism (4 DEGs) and starch and sucrose metabolism (6 DEGs). Up-regulated genes of the susceptible tomato line were involved in the regulation of terpenoid and zeatin (5 and 7 DEGs) biosynthesis, histidine and butanoate (4 and 3 DEGs) metabolism, flavonoids biosynthesis (4 DEGs), and phenylalanine metabolism (4 DEGs). Down-regulated genes were primarily involved in the regulation of ubiquitin-mediated proteolysis (4 DEGs), vesicular transport (2 DEGs), diterpenoid biosynthesis (2 DEGs), DNA replication (2 DEGs), and plant hormone signal transduction (4 DEGs), as well as tryptophan metabolism, flavonoid biosynthesis, starch and sucrose metabolism, pyruvate metabolism, and plant-pathogen interaction (1 to 2 DEGs).

Seven and six DEGs were annotated in the plant-pathogen interaction pathway in resistant and susceptible tomato lines, respectively, with a total of 11 unique DEGs (Fig. S1A). Among them, *CNGC* (*Solyc02g086980.3* and *Solyc05g050380.4*), *EIX1/2* (*Solyc07g008620.1*), *FLS2* (*Solyc02g031790.3*, *Solyc02g068820.3*, and *Solyc02g070890.3*), and *WRKY25/33* (*Solyc09g014990.4*) were up-regulated in the resistant tomato line after Rs inoculation. *CaM*/*CML* (*Solyc04g058170.1*), *FLS2* (*Solyc02g068820.3*), *PIK1* (*Solyc06g075550.3*), and *RPM1* (*Solyc01g073985.1*) were up-regulated, while *CNGC* (*Solyc05g050380.4* and *Solyc09g007840.3*) was up- or down-regulated in the susceptible line after inoculation.

Twelve and eight DEGs were annotated in plant hormone signaling pathways in resistant and susceptible tomato lines, respectively, for a total of 16 unique DEGs (Fig. S1B). Among them, *PYR*/*PYL* (*Solyc06g050500.2* and *Solyc12g095970.3*), *PP2C* (*Solyc06g051940.4*), and *TGA* (*Solyc04g011670.3*) were up-regulated, while *AUX1* (*Solyc10g055260.2*), *SAUR* (*Solyc04g081250.1*), *CRE1* (*Solyc04g008110.3*), and *A-ARR* (*Solyc06g048600.3*) were down-regulated. In addition, *AUX*/*IAA* (*Solyc07g008020.3* and *Solyc03g121060.4*) and *AHP* (*Solyc11g070150.2* and *Solyc01g098400.3*) were up- or down-regulated in the resistant tomato line after Rs inoculation. *PYR*/*PYL* (*Solyc06g050500.2* and *Solyc10g076410.1*) and *JAZ* (*Solyc11g011030.2*) were up-regulated, while *GH3* (*Solyc08g068480.1*), *SAUR* (*Solyc01g110590.4*), and *A-ARR* (*Solyc06g048600.3*) were down-regulated. In addition, *AHP* (*Solyc11g070150.2* and *Solyc01g098400.3*) was up- or down-regulated in the susceptible line after inoculation.

### Two co-expression modules were associated with resistance to Rs

**Fig. 4:**
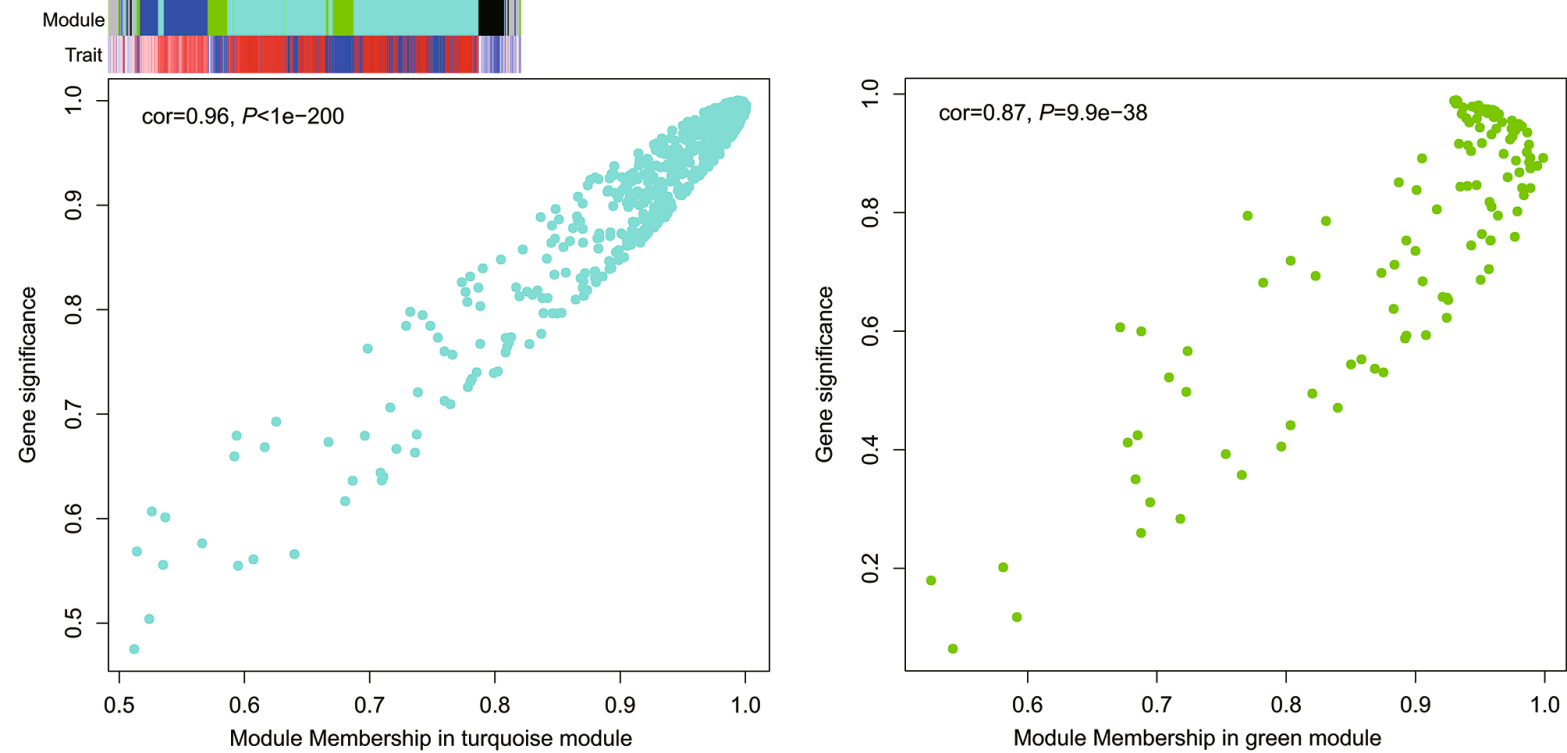
Correlation analysis of genes, co-expression modules, and traits. In the module-trait heatmap, the different colors above represent five modules, while red and blue below represent positive and negative correlations, respectively

Weighted gene co-expression network analysis (WGCNA) resulted in the generation of five co-expression modules, including MEturquoise (567 genes), MEblue (161 genes), MEgreen (119 genes), MEgrey (71 genes), and MEblack (67 genes). After inoculation, significant changes occurred within these co-expression modules, with MEturquoise (cor = 0.99, *P* = 6e-5) and MEgreen (cor=-0.87, *P* = 0.02) showing the highest correlation with resistance phenotype **(**Fig. 4**)**.

In the biological process analysis, the 261 genes in the MEturquoise module were primarily enriched in response to biotic stimulus, oxidation-reduction, defense response, secondary metabolite biosynthesis, steroid metabolism, glutathione metabolism, lipid homeostasis, chitin catabolism, flavonoid biosynthesis, and lignin metabolism. The 46 genes in the MEgreen module were mainly enriched in polysaccharide catabolism and proteolysis, defense response, and oxidation-reduction (Table S2). Meanwhile, the 93 genes in the MEturquoise module were significantly enriched in pathways associated with amino acid metabolism, cutin and wax biosynthesis, flavonoid biosynthesis, fatty acid metabolism, glutathione metabolism, and diterpenoid biosynthesis. Significant association was also found with plant-pathogen interaction, amino sugar and nucleotide sugar metabolism, phenylpropanoid biosynthesis, starch and sucrose metabolism, and plant hormone signal transduction. The 19 genes in the MEgreen module were significantly enriched in glutathione metabolism, alkaloid biosynthesis, amino acid metabolism, plant-pathogen interaction, and amino sugar and nucleotide sugar metabolism (Table S2).

According to the correlation between gene co-expression modules and traits, 251 hub genes derived from the top 150 genes with the highest kME values, including 27 genotype-specific DEGs, were identified in the MEturquoise and MEgreen modules (Table S3). Among them, three resistance genes were found and annotated to function in transport and oxidation. Functional annotations of 24 susceptible genes revealed enrichment in defense response, diterpenoid-like biosynthesis, glutathione metabolism, transport and oxidation, ethylene response, transcriptional regulation, protein modification, RNA processing, and DNA repair (Table S3). Most of these genes were involved in metabolic processes such as biosynthesis, transport, regulation, repair, modification, interaction, and response.

### Core-enriched genes in key pathways were identified

Based on the *q*-value and normalized enrichment score (NES), the top 20 enriched pathways were determined in gene set enrichment analysis (GSEA) (Table S4). Pathways which were common in both resistant and susceptible tomato lines included plant-pathogen interaction, phenylalanine metabolism, DNA and chromatin activity, protein processing, and transport. The tomato lines differed in some metabolic pathways, including regulation of the ribosome, spliceosome, and nucleic acid metabolism. The resistant line positively regulated pathways associated with histidine metabolism, lipid metabolism, and circadian rhythm, while the susceptible line positively regulated pathways related to starch and sucrose metabolism, inositol metabolism, α-linolenic acid metabolism, secondary metabolite biosynthesis, amino sugar and nucleotide sugar metabolism, and fatty acid degradation.

**Fig. 5:**
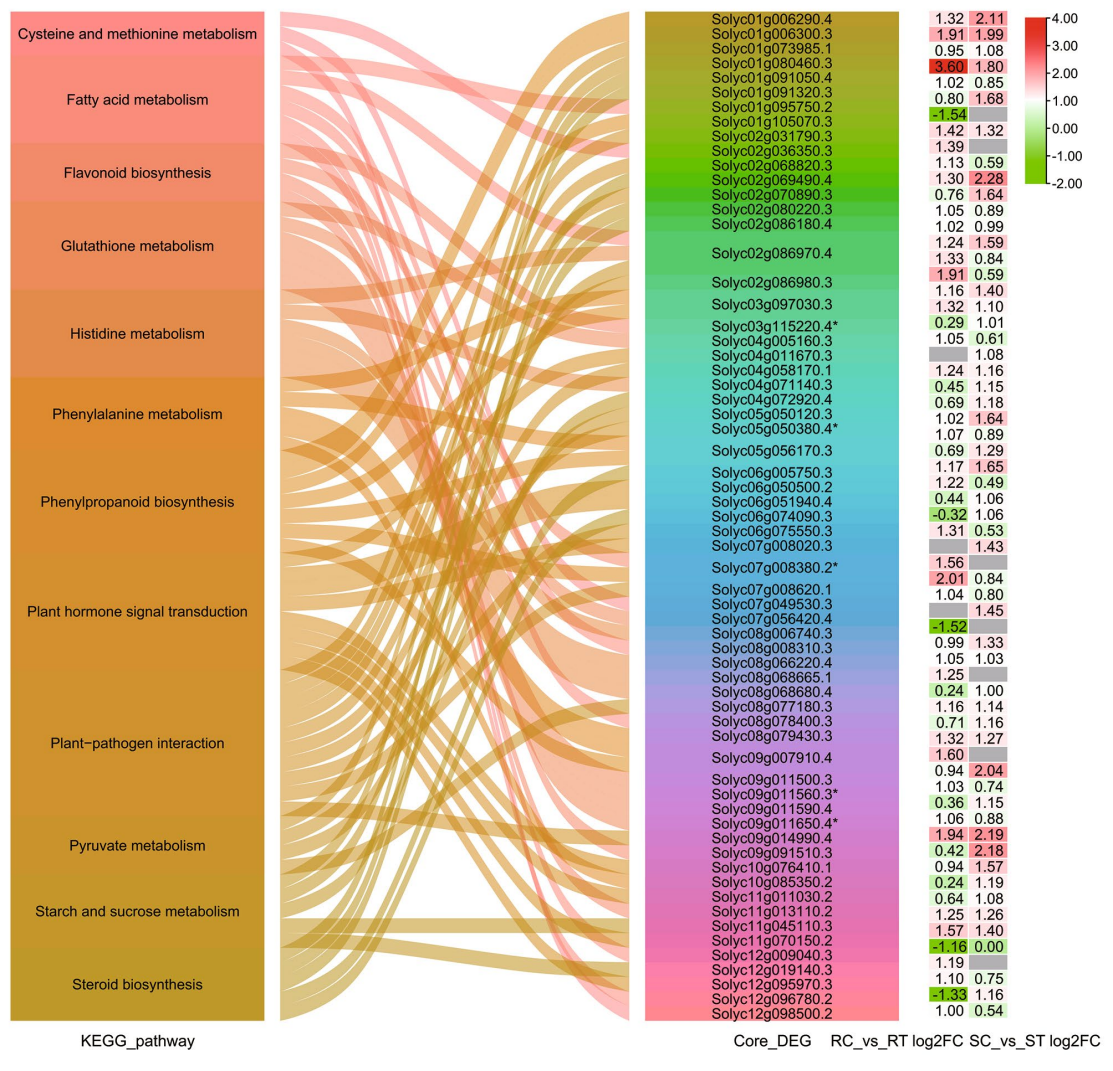
Alluvial relationship between related pathways and core-enriched differentially expressed genes. * represents hub DEGs. The expression of DEGs is shown on the right legend and highlighted with different colors. The color scale is on the right

The numbers of positively regulated and negatively regulated genes were 21 and 25, respectively, in 12 key pathways related to resistance (Table S5). Most of the genes were up-regulated or unchanged with a similar number seen in both tomato lines after inoculation. However, four genes (*Solyc01g095750.2*, *Solyc08g008310.3*, *Solyc12g009040.3*, and *Solyc12g096780.2*) implicated in fatty acid metabolism were down-regulated in the resistant line after inoculation **(**Fig. 5**)**. Among them, *Solyc05g050380.4*, involved in plant-pathogen interaction, *Solyc03g115220.4* and *Solyc07g008380.2*, involved in flavonoid and phenylpropanoid biosynthesis, and *Solyc09g011560.3* and *Solyc09g011650.4*, involved in glutathione metabolism, represented co-expression hubs.

### DEGs with important functions formed a network

A total of 398 (30.34%) DEG-encoded proteins were found to exist in an interaction network. The number of interacting genes ranged from 1 to 135, and *Solyc01g056940.4*, *Solyc03g078630.3*, *Solyc04g082840.4*, *Solyc10g074720.3*, and *Solyc03g083420.4* had more than 100 interactions, including activation, inhibition, binding, catalysis, expression, and reaction. Functional annotation showed enrichment in circadian rhythm, plant-pathogen interaction, plant hormone signal transduction, amino acid metabolism, pyruvate metabolism, fatty acid metabolism, secondary metabolite biosynthesis, protein processing and transport, and DNA and chromatin activity.

**Fig. 6:**
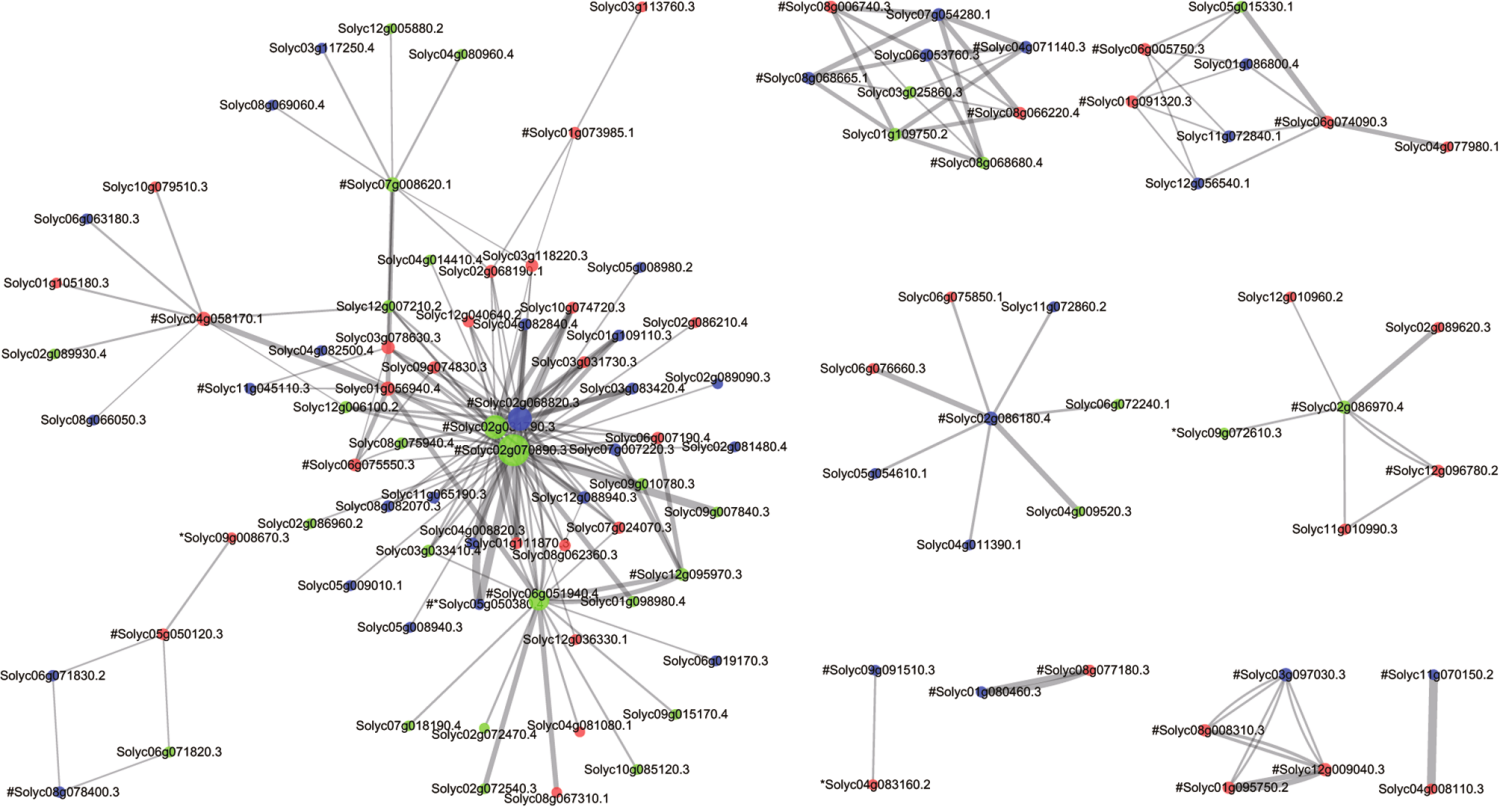
Interaction network (including physical and functional associations) among 32 core-enriched DEGs and 80 others. # and * represent core-enriched DEGs and hub DEGs, respectively. The size of a circle representing a protein indicates the number of other proteins interacting with it. Green, red, and blue indicate positive, negative, and unknown regulation, respectively. The thickness of a gray line indicates the strength of data support for the interaction between two proteins

A total of 32 core-enriched DEGs in 12 critical metabolic pathways related to resistance formed an interaction network with another 80 DEGs (Table S6). *Solyc02g070890.3* involved in plant-pathogen interacted with 40 genes, each 26 for *Solyc02g068820.3* and *Solyc02g031790.3*, and 8–9 for *Solyc07g008620.1* and *Solyc04g058170.1*. *Solyc06g051940.4* involved in plant hormone signal transduction interacted with 17 genes. There were 5–7 interacting genes for *Solyc02g086180.4* and *Solyc06g074090.3* involved in steroid biosynthesis and 5 for *Solyc02g086970.4* involved in fatty acid metabolism **(**Fig. 6**)**. Among the 112 interactions, the positive and negative regulatory genes were 31 and 42, respectively, including 4 hub co-expression genes. *Solyc05g050380.4* was associated with plant-pathogen interaction and was a core-enriched hub DEG, but its expression increased in both resistant and susceptible lines after inoculation.

### Defense and stress response elements were found in multiple DEGs

**Fig. 7:**
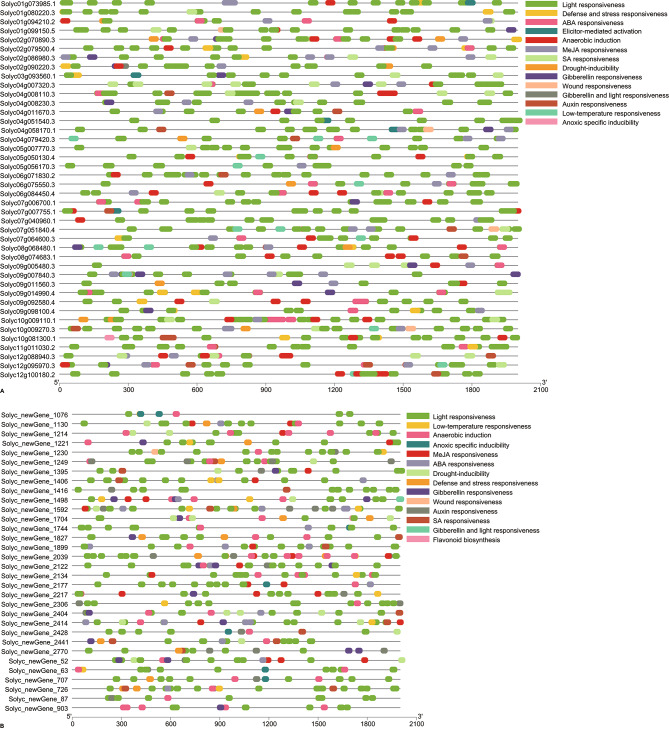
Promoter responsive elements in 74 differentially expressed genes (**A** known genes, **B** new genes). Different colored boxes represent different *cis*-acting elements in the promoter and focus on responses to hormones and stresses

C*is*-acting elements were found in the promoters of 74 DEGs (including 32 new genes) related to transcription initiation (TATA-box and CAAT-box), environmental responsiveness such as defense and stress (W box, TC-rich repeats, and AT-rich sequence), drought (MBS) and low-temperature (LTR), hormones (methyl jasmonate, abscisic acid, salicylic acid, gibberellin, and auxin) and light, and anaerobic induction **(**Fig. 7**)**. Among them, 29 DEGs contained 42 elements related to defense and stress responsiveness.

### Verification of DEGs in response to Rs

**Fig. 8:**
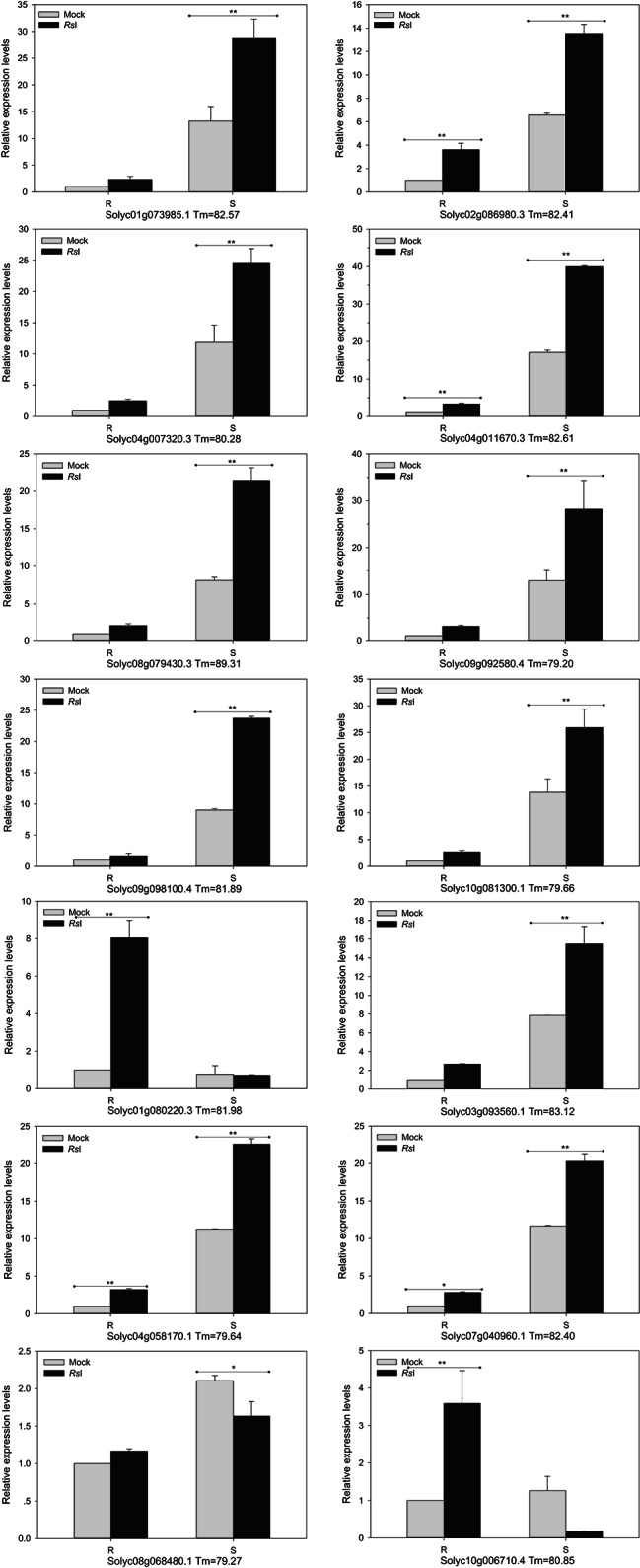
Relative expression levels of 14 vital DEGs in resistant and susceptible tomato lines with *R. solanacearum* infection. Error bar indicates standard deviation. ^*^ and ^**^ indicate statistically significant differences (Tukey’s test, *P* < 0.05 and *P* < 0.01). Tm values indicate the annealing temperature of double chain products for melt curve analysis in qPCR

Expression changes in 62 of the DEGs were verified using RT-qPCR analysis, with 32 showing similar trends upon inoculation, but only 14 of them had statistically significant differences **(**Fig. 8**)**. The relative expression levels of *Solyc02g086980.3*, *Solyc04g011670.3*, and *Solyc04g058170.1* were significantly (*P* < 0.01) up-regulated after inoculation in different tomato lines. They increased by 2.62-, 2.36-, and 2.21-fold in the resistant line, but only 1.06-, 1.34-, and 1.01-fold in the susceptible line, respectively. *Solyc04g007320.3* was significantly (*P* < 0.01) up-regulated in the susceptible line, but the increase was less than that in the resistant line. *Solyc01g073985.1*, *Solyc03g093560.1*, *Solyc08g079430.3*, *Solyc09g092580.4*, *Solyc09g098100.4*, and *Solyc10g081300.1* were significantly (*P* < 0.01) up-regulated by 1.16-, 0.97-, 1.65-, 1.19-, 1.63-, and 0.87-fold in the susceptible line, respectively, but they showed no significant changes in the resistant line. *Solyc07g040960.1* was significantly up-regulated in susceptible (*P* < 0.01) and resistant (*P* < 0.05) lines. *Solyc08g068480.1* was significantly down-regulated in the susceptible line (*P* < 0.05), but was unchanged in the resistant line. *Solyc01g080220.3* and *Solyc10g006710.4* were significantly (*P* < 0.01) up-regulated by 7.04- and 2.59-fold in the resistant line but were unchanged in the susceptible line. Based on these results, *Solyc01g080220.3*, *Solyc02g086980.3*, *Solyc04g011670.3*, *Solyc04g058170.1*, *Solyc08g068480.1*, and *Solyc10g006710.4* may be involved in the resistance response, while *Solyc01g073985.1*, *Solyc03g093560.1*, *Solyc07g040960.1*, *Solyc08g079430.3*, *Solyc09g092580.4*, *Solyc09g098100.4*, and *Solyc10g081300.1* may be opposite.

## Discussion

Bacterial wilt caused by Rs is a major soil-borne disease that significantly reduces tomato production. In this study, high-throughput sequencing was conducted on the transcriptomes of resistant and susceptible tomato inbred lines inoculated with Rs. A total of 35,371 genes were analyzed, including 1,296 new genes. Differential expression analysis revealed a total of 1,312 DEGs, including 836 genotype-specific DEGs. These findings provide a foundation to identify genes associated with resistance response for further functional characterization.

### Multiple key differentially expressed genes were identified in tomato response to Rs

More than 5.73 Gb of high-quality reads in each library were obtained in this study. The mapping rate was greater than 95%, indicating that there was no contamination in the libraries, and the downstream analysis was reliable **(**Table 1**)**. Analysis of the correlation of biological replicates indicated that the results were reproducible (Pearson correlation coefficient > 0.9), and samples were found to cluster primarily based on inoculation status. More than 90% of the 35,371 genes were functionally annotated, and the number was higher than previously seen in the ‘Heinz 1706’ line, possibly due to genotypic differences [7]. A total of 1,296 new genes were discovered, indicating that application of RNA-seq is useful to identify previously unannotated genes. A total of 1,312 DEGs were identified between mock and Rs inoculation, including 836 genotype-specific DEGs **(**Fig. 1 and Table S1). Previous studies have shown that the early defense against bacterial wilt is much stronger in root tissue compared to aboveground tissues, but plant-wide differential expression increases dramatically upon onset of wilting symptoms [8]. Our results further confirmed that significant differential expression took place upon infection with Rs, with changes seen in metabolic pathways, like pattern and auxin response. The number of DEGs in the resistant line was more than that in the susceptible line, including significantly more down-regulated genes, which may indicate that the resistance response is dependent on down-regulation of multiple processes after inoculation. Unique DEGs in resistant or susceptible lines could be used to identify candidate disease resistance genes.

Pathogen infection can initiate expression changes in multiple metabolic pathways. GO annotation of the DEGs indicated that synergistic changes in a series of biological processes, such as stimulus-response, DNA and chromatin activity, proteolysis, secondary metabolism, and defense and immune response, were induced through molecular functions such as transcription regulation, signal transduction, transport, binding and catalytic, and antioxidant activities, to resist pathogen invasion and colonization **(**Fig. 3A**)**. Down-regulated genes performed similar biological processes in the different tomato lines, while the functions of up-regulated genes differed in resistant and susceptible lines. KEGG analysis showed that the DEGs were mainly enriched in metabolic pathways such as plant-pathogen interaction, plant hormone signal transduction, amino acids (histidine, cysteine, methionine, phenylalanine, and tryptophan) metabolism, fatty acid metabolism, starch and sucrose metabolism, glutathione metabolism, secondary metabolite biosynthesis, and plant circadian rhythm **(**Fig. 3B**)**. This enrichment indicated that many of these pathways play important regulatory roles in tomato response to Rs. In addition to differential regulation of metabolic pathways, DNA and chromatin activities, protein processing and transport, and plant circadian rhythm all play important roles in tomato bacterial wilt resistance. Genes which were down-regulated in response to Rs infection in the resistant tomato line were mainly associated with cell wall specialization and secondary metabolism.

Analysis of gene enrichment and co-expression revealed several genes associated with important metabolic pathways related to resistance that may play critical roles in the interaction between tomato and Rs **(**Figs. 5 and 6**)**. A total of 62 DEGs with potentially important functional annotations were assessed via RT-qPCR. These genes had several elements in their promoters which are associated with stress response. Over half of the genes assessed via RT-qPCR showed patterns which were consistent with the transcriptomics data. *Solyc01g080220.3* (β-D-glucanase), *Solyc02g086980.3* (ion channel in plant-pathogen interaction), *Solyc04g011670.3* (transcription factor in plant hormone signal transduction), *Solyc04g058170.1* (calcium-binding protein in plant-pathogen interaction), *Solyc08g068480.1* (amido synthetase in plant hormone signal transduction), and *Solyc10g006710.4* (serine/threonine protein kinase) could all play important roles in the resistance response. *Solyc01g073985.1* (NLR disease resistance protein in plant-pathogen interaction), *Solyc03g093560.1* (ethylene-responsive transcription factor), *Solyc07g040960.1* (salt responsive protein), *Solyc08g079430.3* (amino acid secondary metabolism), *Solyc09g092580.4* (cytochrome P450 like), *Solyc09g098100.4* (NLR resistance protein), and *Solyc10g081300.1* (metacaspase in programmed cell death) were all specific to the susceptible line and may therefore represent susceptibility factors **(**Fig. 8**)**. Our results indicate that DNA repair, transcription and binding, ion transport, signal transduction, pathogenic polysaccharide catabolism, protein phosphorylation, and secondary metabolite biosynthesis are all important components in tomato response to Rs. The overall correlation between RNA-seq and qPCR was not strong (*r* > 0.5 of 13 genes), and such differences are often found when attempting to reproduce transcriptomics analysis via qPCR. Gene expression changes are required to regulate the balance between plant growth and development and response to environmental stress. Overexpression of R genes can enable plants to combat pathogens, but often results in reduced growth. In this study, multiple possible negatively regulated genes were identified. Due to the high conservation of susceptible genes (S genes), a single S gene mutation can be an effective strategy to achieve a broad spectrum and lasting resistance in different crops compared to R gene stacking. However, S genes may have pleiotropic effects and need to be evaluated in application [9].

### DEGs synergistically participated in resistance-related pathways in tomato‑Rs interaction

Plant-pathogen interaction involves both pattern-triggered immunity (PTI) and effector-triggered immunity (ETI). When plants perceive extracellular pathogen-associated molecular patterns (PAMPs), Ca^2+^ concentration increases, calcium-dependent protein kinase (CDPK) and calmodulin (CaM/CML) are activated, and signals are transmitted to NADPH oxidase (RBOH) and nitric oxide synthase (NOS), causing reactive oxygen species (ROS) and NO production, hypersensitive response (HR), cell wall reinforcement, and stomatal closure [10]. When bacterial flagellin is recognized by *FLS2*, the signal is transmitted intracellularly through endocytosis, activating downstream mitogen-activated protein kinases. After the signal reaches the nucleus, WRKY activates or inhibits defense-related genes [11]. In this study, the expression of the *CNGC* gene (*Solyc02g086980.3*) was up-regulated in the resistant tomato line after inoculation. *Solyc05g050380.4* was up-regulated in both resistant and susceptible lines, and *Solyc09g007840.3* was down-regulated in the susceptible line. The expression of the *CaM*/*CML* gene (*Solyc04g058170.1*) was shown to be increased by increases in Ca^2+^ concentration. The expression of *FLS2* genes (*Solyc02g031790.3* and *Solyc02g070890.3*) was up-regulated in the resistant tomato line, and *Solyc02g06882.3* was up-regulated in both resistant and susceptible lines, potentially leading to activation of the downstream *WRKY25/33* gene (*Solyc09g014990.4*) in the resistant line (Fig. S1A). After phosphorylation, *RIN4* has been shown to bind to the R genes *RPM1* and *RPS2* to trigger the HR response. *RPS5* has also been demonstrated to trigger an immune response by binding to *PBS1* [10, 12]. In this study, the expression of the *RPM1* gene (*Solyc01g073985.1*) and *PIK1* gene (*Solyc06g075550.3*) increased in the susceptible tomato line after inoculation (Fig. S1A). This work has indicated that tomato plants can recognize Rs-associated molecular patterns through RLKs located on the cell membrane, which then trigger a change in ion concentration and endocytosis to transmit defense signals, and activate the expression of WRKY transcription factors and defense-related genes in the nucleus, resulting in horizontal resistance. Overall, the resistance response is extremely complicated, and the rapid evolution of the bacterial wilt pathogen makes achieving durable resistance challenging.

In the face of pathogen invasion, plants can activate the expression of resistance genes through signaling molecules such as salicylic acid (SA), jasmonic acid (JA), and ethylene. SA pathway genes positively regulate resistance by participating in phenylalanine metabolism, while JA pathway genes negatively regulate resistance by participating in α-linolenic acid metabolism (Fig. S1B). However, Wu et al. [13] recently found that phenylalanine could not synthesize SA (2-Hydroxybenzoic acid, 2-HBA), but synthesized 4-hydroxybenzoic acid (4-HBA), an isomer of SA. In this study, several DEGs were associated with signaling pathways such as JA/ethylene, SA, auxin, abscisic acid (ABA), and cytokinin (CTK), and JA may play a key role via negative regulation of α-linolenic acid metabolism. French et al. [2] previously demonstrated the importance of the auxin signaling pathway in tomato bacterial wilt resistance. Zou et al. [14] also found that the auxin-responsive gene *ScGH3-1* negatively regulated bacterial wilt resistance in transgenic tobacco, while *GH3* in this study was a positive regulatory gene. Zhou et al. [15], Sanchez-Vallet et al. [16], and Lim et al. [17] reported that the ABA signaling pathway was associated with tobacco resistance to bacterial wilt. Moreau et al. [18] found that CTK played a positive role in the interaction between *Medicago truncatula* and Rs. Yang et al. [19] found that *trans*-zeatin mediates bacterial wilt immunity of pepper and other Solanaceae crops under high temperature and high humidity by regulating chromatin remodeling caused by GST methylation changes. Taken together, these results highlight the complexity of the association between hormones and plant resistance responses.

Other functional molecules identified, such as pathogenesis-related protein (PR), detoxification-like proteins, lignin, lectin, and phytoalexin, have all been shown to be involved in plant disease resistance [20, 21]. Zhang et al. [22] found that SA enhanced disease resistance by inducing the expression of PR genes. Additionally, MYB transcription factors can regulate secondary metabolite biosynthesis to protect plants against biotic stress. For example, *AtMYB15* was shown to regulate defense-induced lignification and basal immunity in *Arabidopsis thaliana* [23]. The expression of *AtMYB11* activated phenylpropanol biosynthetic genes, leading to flavonoid and chlorogenic acid accumulation in tobacco and tomato, and *AtMYB11* and *AtMYB12* regulated flavonoid and caffeoylquinic acid biosynthesis [24]. *AtMYB46*, *AtMYB58*, and *AtMYB63* activated key genes in lignin biosynthesis during secondary wall formation [25, 26]. Additionally, *PpNAC1* plays a major role in phenylalanine biosynthesis and regulation [27]. In this study, we explored the transcriptome dynamics of resistant and susceptible tomato lines upon Rs inoculation and identified several key DEGs that may play direct or indirect roles in the regulation of tomato immune responses. Combined with gene enrichment, interaction and expression analysis, several candidate genes are preferred for subsequent studies with functional genomics approach.

## Conclusion

A total of 75.02 Gb of clean reads were obtained after mRNA sequencing of 12 samples, with at least 5.73 Gb of clean data obtained for each sample. These reads were used to discover 1,296 new tomato genes and conduct differential expression testing that identified 1,312 DEGs. There were 836 genotype-specific DEGs, including 27 co-expression hub genes. More than 98% of the DEGs were functionally annotated in biological pathways, such as DNA and chromatin activity, plant-pathogen interaction, plant hormone signal transduction, secondary metabolite biosynthesis, and defense response. Among core-enriched genes in the 12 pathways related to resistance, 36 genotype-specific DEGs were identified. These genes and their modification factors play a significant role in the interaction between tomato and Rs. Several important functional genes were discovered, which were primarily negatively regulated during defense response. Taken together, the results of our gene expression analysis provide a foundation to further explore tomato resistance responses and provide a resource for the genetic improvement and molecular breeding of tomato.

## Methods

### Plant materials and stress treatment

AH13112111 and G149351121, which were identified as highly resistant (HR) and highly susceptible (HS) to bacterial wilt, respectively, were high-generation tomato inbred lines bred by the Wenzhou Academy of Agricultural Sciences (Wenzhou, China) [28]. The seeds of two tomato lines were sterilized, rinsed in sterile water, and sown in pots containing a mixed matrix. On the appearance of the fourth leaf, homogeneous seedlings were infected with 10^8^ colony-forming units (CFUs)/mL of Rs (GenBank No. KJ913693) by the root inoculation method. The control seedlings were mock-inoculated with distilled water. Next, the seedlings were incubated in a culture chamber under controlled conditions (14 h/10 h diurnal cycle, 28/25°C day/night temperature, and 80% relative humidity). After 48 h, seedlings were sampled, quickly frozen in liquid nitrogen, and stored at -80 °C for RNA isolation. Three biological replicates were set.

### Quality control and processing of sequencing data

Total RNA was extracted using the plant Trizol extraction kit (Sangon Biotech; Shanghai, China) according to the manufacturer’s instructions. The RNA concentration, purity, and integrity were assessed using a NanoDrop system. After mRNA sequencing libraries were constructed and qualified, paired-end sequencing was performed using an Illumina HiSeq2500 high-throughput platform (Biomarker Technologies; Qingdao, China). Clean reads were obtained after removing reads with adapter contamination and low-quality bases (percent of N base is greater than 10% or percent of low-quality bases greater than 50%). The remaining reads were then mapped against the tomato reference genome (ITAG4.0) and quantified using HISAT2 [29] and StringTie [30].

### Screening and annotation of differentially expressed genes

Transcript and gene expression levels were calculated using the maximum flow algorithm of StringTie [30] and normalized using fragments per kilobase of transcript per million fragments mapped (FPKM). Pearson’s correlation coefficient was used as a common evaluation index to determine the correlation between biological replicates. DEGs were considered significant when they showed a FC ≥ 2 and had a FDR < 0.01 when using an adjusted *p*-value from the Benjamini-Hochberg method in DESeq2 [31].

Genes were annotated by a blast alignment (E ≤ 1e-5) using the COG (cluster of orthologous groups of proteins, http://www.ncbi.nlm.nih.gov/COG/), GO (gene ontology consortium, http://www.geneontology.org/), KEGG (Kyoto encyclopedia of genes and genomes, http://www.genome.jp/kegg/), KOG (eukaryotic orthologous groups, http://www.ncbi.nlm.nih.gov/KOG/), NR (non-redundant protein database, https://ncbi.nih.gov/blast/db/), Pfam (http://pfam.xfam.org/), and Swiss-Prot (swiss-prot protein database, http://www.uniprot.org/) databases.

### Gene co-expression network analysis

Weighted gene co-expression network analysis was performed using the WGCNA R package [32]. A soft-threshold of co-expression network clustering was selected with R^2^ > 0.9 as the standard. All FPKM values were transformed into a topological overlap matrix (TOM) for gene hierarchical clustering. Different genes were classified into separate co-expression modules by the dynamic tree-cut method. Genes with a kME (eigengene connectivity) > 0.7 were grouped into modules with a minimum of 30 genes per module. Parallel modules in clustering were combined, and the correlation between different modules and the module membership was calculated using 0.25 as the boundary.

### Gene set enrichment analysis

Gene sets in the KEGG pathway and biological process, cellular component, and molecular function of GO classes were extracted to perform GSEA. Enrichment of the gene set was scored as the log_2_FC value, with a *p*-value < 0.001 or FDR < 0.05. Alluvial between pathways and genes was plotted by Bioinformatics (http://www.bioinformatics.com.cn/), an online platform for data analysis and visualization.

### Protein interaction analysis

The interactive proteins were located by STRING11 (https://string-db.org/cgi/input.pl), and the regulatory network was constructed by Cytoscape [33].

### Promoter ***cis***-acting element analysis

The 2000 bp upstream sequences of DEGs in tomato were downloaded from Solanaceae Genomics Network (https://solgenomics.net/), and the *cis*-acting regulatory elements in these promoters were analyzed by the PlantCARE database (http://bioinformatics.psb.ugent.be/webtools/plantcare/html/).

### Quantitative expression following ***Rs***I

RT-qPCR was performed using the SYBR Green PCR Master Mix on StepOne Plus System (ABI; CA, USA) to validate the results of RNA sequencing. After extracting the total RNA, single-stranded cDNA was synthesized using the Maxima Reverse Transcriptase kit. The reaction mixture contained 2 µL of cDNA, 0.4 µL of each sequence-specific primer, 10 µL of SYBR Green, and 7.2 µL of ddH_2_O. The PCR reaction conditions were 45 cycles of denaturation at 95 °C for 5 s and annealing for 30 s and extension at 60 °C. The *SlRPL2* gene was used as an internal control. All primers were designed using Primer Premier 5.0 (Table 2). The values of relative gene expression were calculated using the 2^−ΔΔCt^ method.

**Table 2:** Primers used for RT-qPCR

Gene	Forward primer (5’-3’)	Reverse primer (5’-3’)
*SlRPL2*	GTCATCCTTTCAGGTACAAGCA	CGTTACAAACAACAGCTCCTTC
Solyc01g073985.1	GTTGGCTCGTGGAGGATGAT	TGCTGGCTGCTTCACTTCA
Solyc01g080220.3	GCAGGAGCACAATGTATGAAGAATC	TCAGGAACAACCACCAAGTACC
Solyc02g086980.3	GCGTTGAGTTAGGCAGTAAGG	CTAGAAGATGATCCAGTGAGTGAGA
Solyc03g093560.1	CGTTGAGATTGAGAAGAAGCATTAC	CCGCATCCACAGCAGTATC
Solyc04g007320.3	TATGCTGAATCGTCGTGGTGTT	GCTTCCTCTAGTGCTCCTCTC
Solyc04g011670.3	CATTATCGTTGATGGTTGCCTGAA	GCGTTCTGCTGATGTCTTCC
Solyc04g058170.1	GCCTTGTGTGTCTTGATGAGTT	TCCTCGTGCTTATAATTGCTACCT
Solyc07g040960.1	TTACGCAAGAGAACAGAAGTGAAG	GCAAGAGTTGGTGAGCAATCT
Solyc08g068480.1	ATCCATTGGTCCATTAGAGTTATGC	AGTGCATCTAGGAGTCTTGAATTGA
Solyc08g079430.3	CGGAGAAGGAGGTAGTGTTGAA	GGCAGAAGTGGCGGAGAT
Solyc09g092580.4	CAAGCCGCCACTTCACAA	GCCAACATTTCTTCCGTCACT
Solyc09g098100.4	GCGAACAAGTCAAGTGCCATAC	TTCACCTCATTCCACCAAGATTCT
Solyc10g006710.4	AGTGGTTGGAACATACGGTTACAT	GAGGTTGAGATTGTGGTCTGGAT
Solyc10g081300.1	TCCAAGCCTAAATTCATCCCACAT	TCCTCCAACATCTTCACATTCTTCA

## Electronic supplementary material

Below is the link to the electronic supplementary material.

Additional file 1: Fig. S1. KEGG enrichment of differentially expressed genes in the plant-pathogen interaction (A) and plant hormone signal transduction (B) pathways

Additional file 2: Table S1. Distribution of differentially expressed genes in resistant and susceptible tomato lines

Additional file 3: Table S2. GO_BP and KEGG enrichment of genes in the Meturquoise and MEgreen modules

Additional file 4: Table S3. Annotations of hub differentially expressed genes in the MEturquoise and MEgreen modules

Additional file 5: Table S4. The top 20 GSEA pathways of genes in resistant and susceptible tomato lines

Additional file 6: Table S5. Annotations of core-enriched differentially expressed genes in key pathways

Additional file 7: Table S6. Prediction and expression of 32 core-enriched interacting DEGs

## Data Availability

The datasets generated during the current study are available in the NCBI repository, www.ncbi.nlm.nih.gov/sra/?term=PRJNA902097.

## Abbreviations

*Rs*I: *Ralstonia solanacearum* inoculation
HR: Highly resistant
HS: Highly susceptible
RLP: Receptor-like protein
RLK: Receptor-like kinase
NBS-LRR: Nucleotide binding site-leucine rich repeat
RNA-seq: RNA sequencing
FC: Fold change
FDR: False discovery rate
DEG: Differentially expressed gene
BP: Biological process
CC: Cellular component
MF: Molecular function
WGCNA: Weighted gene co-expression network analysis
GSEA: Gene set enrichment analysis
PTI: Pattern-triggered immunity
ETI: Effector-triggered immunity.
